# ASSIST Applicability Scoring of Surgical trials. An Investigator-reported aSsessment Tool

**DOI:** 10.1371/journal.pone.0042258

**Published:** 2012-08-15

**Authors:** Idriss Tourabaly, Isabelle Boutron, Rémy Nizard, Philippe Ravaud

**Affiliations:** 1 Inserm, U738, Paris, France; Assistance Publique des Hôpitaux De Paris, Hôpital Hôtel Dieu, Centre d'épidémiologie Clinique, Paris, France; University Paris Descartes, Sorbonne Paris Cité, Faculté de Médecine, Paris, France; 2 Assistance Publique des Hôpitaux de Paris, Hôpital Lariboisière, Service de Chirurgie Orthopédique et Traumatologique, Paris, France; University Paris Diderot, Sorbonne Paris Cité, Faculté de Médecine, Paris, France; Research and Development Corporation, United States of America

## Abstract

**Context:**

We aimed to develop a new tool for assessing and depicting the applicability of the results of surgical randomized controlled trials (RCTs) from the trial investigators' perspective.

**Methods:**

We identified all items related to applicability by a systematic methodological review, and then a sample of surgeons used these items in a web-based survey to evaluate the applicability of their own trial results. For each applicability item, participants had to indicate on a numerical scale that was simplified as a three-item scale: 1) items essential to consider, 2) items requiring attention, and 3) items inconsequential to the applicability of the results of their own RCT to clinical practice. For the final tool, we selected only items that were rated as being essential or requiring attention for at least 25% of the trials evaluated. We propose a specific process to construct the tool and to depict applicability in a graph. We identified all investigators of published and registered ongoing RCTs assessing surgery and invited them to participate in the web-based survey.

**Results:**

148 surgeons assessed applicability for their own trial and participated in the process of item selection. The final tool contains 22 items (4 dedicated to patients, 5 to centers, 5 to surgeons and 8 to the intervention). We proposed a straightforward process of constructing the graphical tool: 1) a multidisciplinary team of investigators or other care providers participating in the trial could independently assess each item, 2) a consensus method could be used, and 3) the investigators could depict their assessment of the applicability of the trial results in 4 graphs related to patients, centers, surgeons and the intervention.

**Conclusions:**

This investigator-reported assessment tool could help readers define under what conditions they could reasonably apply the results of a surgical RCT to their clinical practice.

## Introduction

In surgery, the results of a randomized controlled trial (RCT) cannot be relevant to all patients and all settings [1], and the intervention should probably not be performed by all surgeons, whatever their expertise [2]. Consequently, transposing the results of research to clinical practice requires an adequate assessment of the applicability of trial results, also named external validity, or generalizability [3]. Assessing applicability supposes determining to whom, how and under what conditions the results of the trial should be applied.

However, assessing the applicability of trial results from the published article is challenging, if not impossible. In fact, applicability is a complex and multidimensional concept [4],[5],[6] and depends on participant recruitment and characteristics, participating centers and surgeons, and how all the different components of the intervention were planned and actually implemented. Therefore, determining from only the content of a report how an intervention should be implemented in clinical practice is difficult. Indeed, descriptions in journal articles may obscure some aspects of the interventions performed in the trial. Indicating in the text of a report the difficulties of some technical aspects of the procedure may not be possible. Similarly, the text of the report may not adequately convey the complexity of pre-operative and post-operative care in a surgical trial. Assessing applicability implies knowing exactly how the study was actually conducted, which can be extremely difficult for someone who did not participate in the trial. Further, despite reporting guidelines aimed at improving transparency [7], data necessary to appraise applicability are lacking in most reports [8], [9], [10]. Therefore, consistently determining the applicability of the results of a trial assessing surgical procedures only from the publication is problematic.

To overcome these important issues, we propose a new paradigm. Our hypothesis is that investigators and healthcare providers who participate in a trial are in an important position to appraise the applicability of their trial results, that is, determine for whom and how the intervention could be implemented in clinical practice. Consequently, instead of focusing only on improving transparency in published reports, we propose a tool to allow for an “investigator-reported assessment and depiction" of applicability.

## Materials and Methods

The new tool aims at assessing and depicting the applicability of the results of a trial from the trial investigators' perspective. We focused on surgical trials. The tool is an investigator-reported assessment of the applicability of trial results. The investigators assess and depict the conditions related to the patients, centers, surgeons and the intervention that need consideration from their point of view before applying the results of their surgical RCT in clinical practice.

To develop the tool, we identified all items related to applicability relevant to medical and surgical trials by a systematic methodological review, and then a sample of surgeons used these items in a web-based survey to evaluate the applicability of their own trial results.

### Identification of relevant applicability items

We performed a systematic review of articles in Medline via PubMed and the Cochrane Methodology Register to identify the most relevant items for applicability. The search strategy is available in the text S1. The titles and abstracts of retrieved citations were screened by one of us (I.T.).

An article was included if the study was published in English and was identified as a methodological study assessing applicability or a study assessing any treatment and discussing applicability.

From all selected articles, one of us (I.T.) extracted all items of applicability relevant to medical and surgical trials. Among the domains affecting applicability [7], [11], [12] (patients, intervention, comparators, centers, surgeons and outcomes), we selected only items related to patients, centers, surgeons and the intervention. We did not consider the domains of applicability related to choosing the outcome and the comparator because this judgment can probably be better evaluated by the reader and do not depend on the actual conduct of the trial. Two authors (IT, IB) excluded duplicate items and synonymous terms and classified all items into domains (patients, surgeons, centers and the intervention). Then, we reworded the items to build the questionnaire. After that, the questionnaire was pilot tested by a panel of surgeons and methodologists to revise questions for clarity.

### Identification of participants

We aimed to identify authors of published RCT results and investigators of ongoing RCTs.

We searched Medline via PubMed using the following strategy (((randomized controlled trial AND (Humans[Mesh] AND Randomized Controlled Trial[ptyp] AND (English[lang]))) AND surgery) AND “2009"[Publication Date]: “2010"[Publication Date]. The limits were: Humans, Randomized Controlled Trial, and English. We selected only RCTs assessing a surgical procedure published in a journal indexed on ISI Web of Science between January 1, 2009 and February 15, 2010. Exclusion criteria were nonrandomized controlled trials, trials assessing other treatments such as pharmacological treatments, rehabilitation, education, etc., and cost-effectiveness trials.

We also searched for all ongoing RCTs assessing a surgical procedure through the International Clinical Trials Registry Platform Search Portal of the World Health Organization at http://apps.who.int/trialsearch/AdvSearch.aspx on April 5, 2010. Then, we systematically extracted the names of all authors and investigators of the selected RCTs and searched for their e-mail addresses. For RCTs indexed in PubMed, we extracted the e-mail addresses from 1) the abstract of the report in PubMed; 2) the full text, if available, or the journal website; or 3) publications in which authors were the corresponding authors of a report of another trial by using the Pubmed advanced search with the search builder [First Author].

For ongoing RCTs, we extracted the investigators' e-mail addresses from the clinical trial register, and if not available, we systematically searched for publications in which investigators were the corresponding authors of another trial by using the Pubmed advanced search with the search builder [First Author].

### The web-based survey

All authors and investigators were invited by e-mail to participate in a web-based survey about the applicability of the results of their own trial in clinical practice. They received a personal login to the Web site (http://www.nonpharmacological.com/Survey/Page1.php), and if they agreed to participate, they completed the online questionnaire we developed (available upon request).

The web survey was built on 4 pages, one page dedicated to each domain: 1) patients, 2) centers, 3) surgeons and 4) the intervention. Two reminders to complete the survey were sent by e-mail to nonresponders on days 4 and 14.

All questions related to each domain were built on the same pattern:

- Do you think the results of your trial could be transposed to any patients?
- Do you think the results of your trial could be transposed to any center?
- Do you think the results of your trial could be transposed if the intervention were performed by any surgeon?
- What are the essential components of your intervention needed to transpose the results of your trial to clinical practice?

Surgeons could also indicate any other items that were not listed but were deemed important in the context of their own trial. For each item, participants had to answer on a numerical scale [1 to 9] that was then simplified according to the scale tertile in a three-item scoring system: 1) items essential to consider before applying the RCT results to clinical practice; 2) items requiring attention before applying the results to clinical practice; and 3) items inconsequential to applying the results to clinical practice.

The web survey also asked questions about demographic data of participants (age, sex, area of specialty), whether they worked in a university hospital, the number of surgical RCTs they had been involved in, and the number of surgical RCTs for which they were principal investigator.

Answers for authors who completed the survey but were not surgeons (e.g., methodologists, other clinicians, physiotherapists) were excluded because we wanted to have a homogenous sample of participants. Further, if several surgeons participating in the same trial completed the survey, we considered the answer of only 1 of the surgeons by choosing 1) the corresponding author when possible or 2) a randomly chosen author.

Only items rated as being essential or as requiring attention before applying the results to clinical practice for more than 25% of the trials were selected in the final tool.

### Reproducibility in assessing the applicability of results of surgical RCTs

We analyzed the reproducibility of the applicability assessment by 2 surgeons involved in the same trial who completed the survey. If more than 2 surgeons completed the survey, we randomly chose one pair. Therefore, data for only pairs of surgeons were analyzed, apart from missing data, to determine the reproducibility.

### Statistical analysis

Categorical variables are described with frequencies and percentages and quantitative variables with means (standard deviation) or medians (interquartile ranges). We used the rate of agreement with 95% confidence intervals (95% CIs) to determine the level of reproducibility. We defined agreement as a difference of ≤3 rating on the [1 to 9] numerical scale between the 2 investigators. Data analysis involved use of SAS v9.1 (SAS inst., Cary, NC).

### Ethics

Of note, this study was conducted in France, and according to French legislation, ethical approval for the study by an ethics committee was not necessary because we did not include any patients and we did not perform any experiments. We only conducted an online survey where participants of this survey were informed of the aim of the survey. We did not explicitly obtain informed consent because we considered that if the participants took time to complete the survey, it implied that they consented to participate in this study.

## Results

### Participants

**Figure 1:**
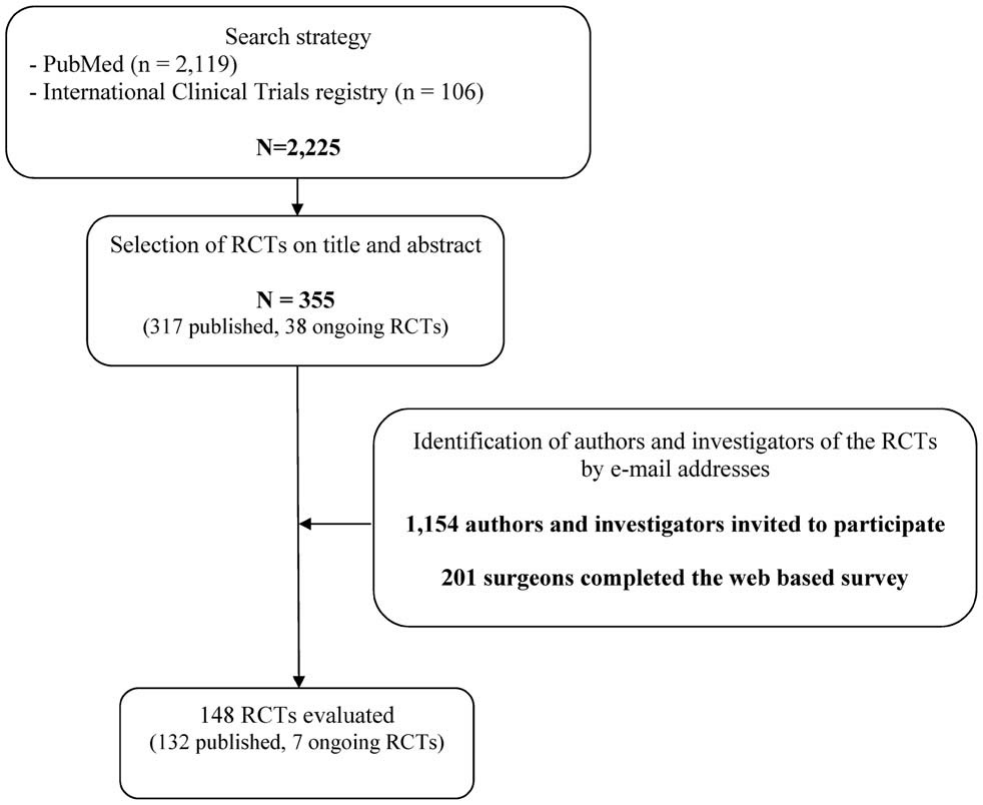
The flow chart of the selection of participants.

The flow chart of participants through the study is in Figure 1. The search strategy identified 2,225 RCTs (2,119 with published results and 106 ongoing): 355 RCTs were eligible for analysis (317 with a full text and 38 ongoing). In total, the survey was completed by at least 1 surgeon for 148 RCTs (132 published RCTs and 7 ongoing RCTs).

Of the 148 surgeons, 86% were male, and the mean (SD) age was 44 (10) years; 72% were working in a university hospital (Table 1). About half of the surgeons (51%) were orthopedic or gastrointestinal surgeons. In total, 95% of surgeons had participated in at least 2 surgical RCTs, and 52% had been principal investigator of a surgical RCT.

**Table 1:** The general characteristics of surgeons completing the web survey (N = 148).

Surgeons' characteristics	N = 148 (%)
Age (years) (mean [SD])	44 [±10]
Sex (male)	73 (86)
Working in university hospital	63 (72)
Number of surgical randomized controlled trials involved in (median [IQR])	5 [3]–[8]
Number of surgical randomized controlled trials involved in as principal investigator (median [IQR])	1 [0–3]
Surgical area:	
Orthopaedic surgery	43 (31)
Gastrointestinal surgery	28 (20)
Urology	14 (10)
Gynecology-Obstetric surgery	10 (7)
Ophtalmology	10 (7)
Otorhinolaryngology	8 (6)
Vascular surgery	8 (6)
Cardio-thoracic surgery	9 (6)
Neuro-surgery	4 (3)
Plastic surgery	4 (3)
Maxillo-facial surgery	1 (1)

### Development of the applicability tool

From the literature search, we selected 25 items for the web-based survey: 7 items were related to patients, 5 to centers, 5 to surgeons and 8 to the intervention (the detailed checklist is available upon request). The results of the survey are summarized in Table 2.

1. **Patients.** For more than 80% of the trials, surgeons did not consider sex, socioeconomic status or ethnicity as affecting the applicability of the results of a surgical RCT. However, the age of patient was considered essential or requiring attention for one-third of the trials and weight, severity of disease, co-morbidities and co-medications for about 40%.
2. **Centers.** For about one-third of the trials, surgeons considered items related to the center's setting, the center's diagnostic facilities, and the country location of the center essential or requiring attention in terms of the applicability of trial results. Items dedicated to the center's surgical volume and medical infrastructure were considered essential or requiring attention for 49% and 41% of the trials, respectively.
3. **Surgeons.** For more than 60% of the reports, all items related to the surgeon were considered essential or requiring attention for the applicability of trial results. Moreover, for one-third of the trials, the surgeon's years in practice and surgeon's volume for the procedure were considered essential; for about 40% of the trials, the surgeon's professional qualification and level of skill were considered essential, and for half of the trials, specific training was essential.
4. **Intervention.** The relevance of items dedicated to the intervention varied. Items such as preoperative care, anesthetic management and intensive care were considered essential for 23%, 19% and 11% of the trials, respectively. Items related to postoperative care and quality of the collaboration were considered essential for 36% and 40% of the trials, and use of specific equipment, standardization of patient management, and follow-up organization were considered essential for more than half of the trials.

**Table 2:** Responses of surgeons to items related to the applicability of their trial results.

	N = 148	Item essential for applicability N (%)	Item requiring attention for applicability N (%)	Item inconsequential for applicability N (%)
**PATIENTS**				
– Age	107	21 (19.6)	12 (11.2)	74 (69.2)
– Sex	108	8 (7.4)	12 (11.1)	88 (81.5)
– Socioeconomic status	113	7 (6.2)	11 (9.7)	95 (84.1)
– Ethnicity	112	9 (8.0)	13 (11.6)	90 (80.4)
– Weight or body mass index	113	13 (11.5)	33 (29.2)	67 (59.3)
– Severity of disease	113	20 (17.7)	25 (22.1)	68 (60.2)
– Co-morbidities or co-medications	113	15 (13.3)	32 (28.3)	66 (58.4)
**CENTERS**				
– Center's setting	142	21 (14.8)	16 (11.3)	105 (73.9)
– Center's surgical volume	110	28 (25.4)	26 (23.6)	56 (50.9)
– Center's medical infrastructure	141	33 (23.4)	25 (17.7)	83 (58.9)
– Center's diagnostic facilities	141	24 (17.0)	21 (14.9)	96 (68.1)
– Country where the center is located	141	18 (12.8)	31 (22.0)	92 (65.2)
**SURGEONS**				
– Surgeon's professional qualification or specific expertise	138	59 (42.7)	34 (24.6)	45 (32.6)
– Surgeon's years in practice	137	43 (31.4)	42 (30.7)	52 (38.0)
– Surgeon's level of skill	139	59 (42.4)	36 (25.9)	44 (31.6)
– Surgeon's volume for the procedure evaluated	137	51 (37.2)	37 (27.0)	49 (35.8)
– Specific training	137	75 (54.7)	29 (21.2)	33 (24.1)
**INTERVENTION**				
– Use of specific equipment	109	57 (52.3)	12 (11.0)	40 (36.7)
– Preoperative care provided	109	25 (22.9)	21 (19.3)	63 (57.8)
– Anesthetic management used	109	21 (19.3)	27 (24.8)	61 (56.0)
– Intensive care treatment provided	108	12 (11.1)	23 (21.3)	73 (67.6)
– Postoperative care provided	109	39 (35.8)	26 (23.8)	44 (40.4)
– Quality of collaboration	107	43 (40.2)	21 (19.6)	43 (40.2)
– Standardization of patient management	108	64 (59.3)	28 (25.9)	16 (14.8)
– Follow-up organization	107	60 (56.1)	27 (25.2)	20 (18.7)

### Reproducibility in assessing the applicability of results of surgical RCTs

We obtained 40 trials for which at least 2 surgeons answered the survey. Table 3 presents the rate of agreement for all items of the survey. The rate of agreement was between 76% and 90% for patients, 68% and 82% for centers, 53% and 68% for surgeons and 58% and 75% for the intervention.

**Table 3:** The reproducibility of evaluating applicability by surgeon authors of the same trial.

Variable	n	Rate of agreement (%) [95% confidence interval]
Age	30	77 [58–90]
Sex	29	90 [73–98]
Socioeconomic status	33	85 [68–95]
Ethnicity	32	84 [67–95]
Weight or body mass index	33	79 [61–91]
Severity of disease	33	76 [58–89]
Co-morbidities or co-medications	33	82 [65–93]
Center's setting	38	82 [66–92]
Center's surgical volume	32	78 [60–91]
Center's medical infrastructure	38	79 [63–91]
Center's diagnostic facilities	38	68 [51–83]
Country where the center is located	38	71 [54–85]
Surgeon's professional qualification or specific expertise	36	60 [35–70]
Surgeon's years in practice	37	60 [42–76]
Surgeon's level of skill	38	53 [36–69]
Surgeon's volume for the surgical procedure evaluated	38	68 [51–83]
Specific training	37	57 [39–73]
Use of specific equipment	31	58 [39–75]
Type of anaesthesia used	32	62 [44–79]
Anesthetic management used	32	75 [57–89]
Intensive care treatment provided	32	75 [57–89]
Postoperative care provided	31	68 [48–83]
Quality of collaboration	32	63 [44–79]
Standardization of patient management	31	61 [42–78]
Follow-up organization	30	63 [44–80]

### Proposal for the investigator-reported graphical tool and process of construction

In the final tool, we selected only items that were rated as essential or requiring attention for at least 25% of the trials. Consequently, the items dedicated to patient sex, socioeconomic status, and ethnicity were excluded. The final tool, provided in Table 4, contains 22 items: 4 dedicated to patients, 5 to centers, 5 to surgeons and 8 to the intervention. Further, investigators did not propose new items that were not in the initial checklist, and no new item was added.

**Table 4:** The final tool.

**Do you think the results of your trial could be transposed to any PATIENTS?**
1. Whatever their **age** (even in elderly)?
2. Whatever their **weight** or **body mass index** (even overweight patients)?
3. Whatever the **severity of their disease** (even patients with severe disease)?
4. Whatever their **co-morbidities** (e.g, diabetes) or **co-medications**?
**Do you think the results of your trial could be transposed to any CENTER?**
5. Whatever the center's **setting** (eg, general hospital, teaching hospital)?
6. Whatever the center's **surgical volume** (even in centers with low surgical volume)?
7. Whatever the center's **medical infrastructure** (eg, rehabilitation unit, intensive care unit, etc.)?
8. Whatever the center's **diagnostic facilities** (eg, availability of diagnostic procedures etc.)?
9. Whatever the **country where the center is located**?
**Do you think the results of your trial could be transposed if the intervention were performed by any SURGEON:**
10. Whatever the surgeon's **professional qualification or specific expertise** (eg, microsurgery, endoscopy)?
11. Whatever the surgeon's **years in practice**?
12. Whatever the surgeon's level of **skill**?
13. Whatever the surgeon's **volume for the surgical procedure evaluated?**
14. Whatever the **specific training “provided"** for the procedure evaluated?
**What are the essential components of your intervention needed to transpose the results of your trial to clinical practice?**
15. The use of **specific equipment** (ie, new instrument, implant used)?
16. The **preoperative care** provided (eg, pharmacological or nonpharmacological treatment)?
17. The **anesthetic management** used (eg, type of anaesthesia, cell saver blood, etc.)?
18. The **intensive care treatment** provided?
19. The **postoperative care** provided (eg, pharmacological or nonpharmacological treatment)?
20. The **quality of collaboration** between surgeons, anaesthesiologists, etc. (eg, commitment, communication)?
21. The **standardization of patient management** (eg, patient information, pain management, mobilization, etc.)?
22. The **follow-up organization** (ie, once a week, once a month, phone recall)?

**Figure 2:**
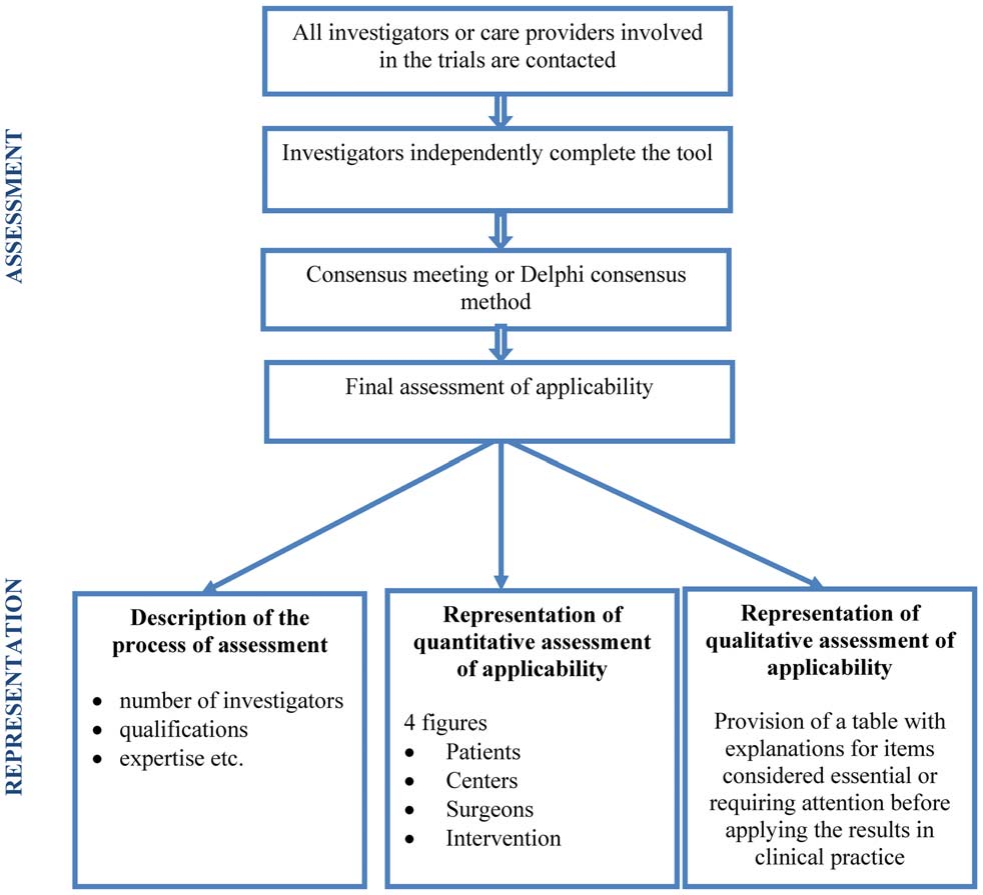
How the tool should be used.

**Figure 3:**
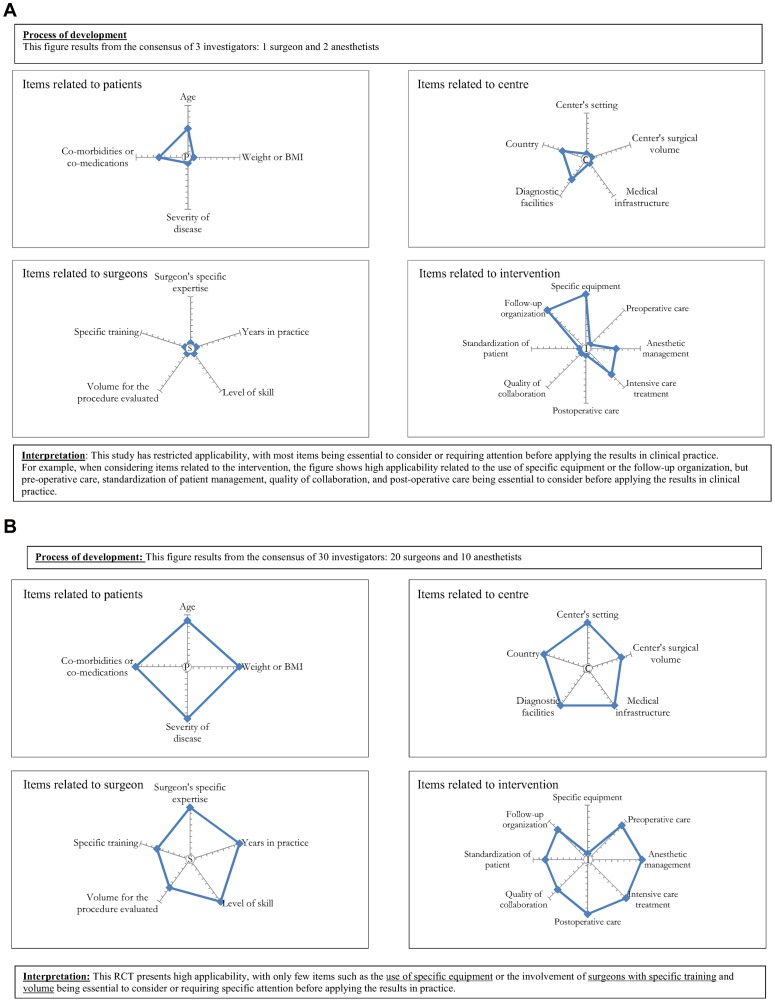
Hypothetical examples of randomized controlled trials. Figure 3a is an example of a trial with restricted applicability: *“Short-term outcomes from a prospective randomized trial comparing laparoscopic and open surgery for colorectal cancer".* Figure 3b is an example of a trial high applicability: *“Cemented versus Uncemented Hemiarthroplasty for Displaced Femoral Neck Fractures"*. The center of the “wheel" represents restricted applicability (1) and the other end of the “wheel" represents high applicability (9). Each axis represents one item rated on a scale from 1 (center of the “wheel") to 9 (other end of the “wheel"). Items scored ≤3 were considered essential to consider before applying the results of the RCT to clinical practice; items scored between 4 and 6 were considered as requiring attention before applying the results to clinical practice; items scored ≥7 were considered as being inconsequential for applying the results to clinical practice.

We propose that the tool should be used as described in Figure 2. In a first step, investigators or other healthcare providers participating in the trial could independently determine for each item whether the results of their trial could be transposed to any patient, any center or any surgeon and to identify components of their intervention they consider essential for the applicability of their trial results. In a specific context, other items considered important for applicability could be added to some domains of the tool. We propose a multidisciplinary assessment (surgeons, anesthetists etc.) to obtain an assessment of the applicability of trial results from different perspectives, not just the surgeon's perspective. Then, in a second step, a consensus method (eg, Delphi consensus method) could be used to achieve consensus on the different assessments of the applicability of the trial results. Finally, the investigators could depict their assessment of the applicability of the trial results in a figure. The results could be summarized in 4 graphs related to 1) patients, 2) centers, 3) surgeons and 4) the intervention. Figure 3a and 3b are examples of the applicability of results of 2 RCTs. The first example is an RCT comparing laparoscopic and open surgery for colorectal cancer with low applicability of results. For example, when considering items related to the intervention, the figure shows high applicability related to the use of specific equipment or the follow-up organization but that the pre-operative care, standardization of patient management, quality of collaboration, and post-operative care are essential to consider before applying the results in clinical practice. The process of developing this tool should clearly appear in the figure. For example, in this case, 1 surgeon and 2 anesthetists involved in the trial achieved consensus. The second example is an RCT comparing cemented versus uncemented hemiarthroplasty for displaced femoral neck fracture. The assessment of applicability was achieved by consensus among 20 surgeons and 10 anesthetists involved in the trial. This RCT presents high applicability, with only a few items such as the use of specific equipment or the involvement of surgeons with specific training and volume being essential to consider and requiring specific attention, respectively, before applying the results in practice. A table with justifications for items considered essential or requiring attention before applying the results in clinical practice could be reported with the figure.

## Discussion

We developed a tool to assess and represent the applicability of results of surgical RCTs from the investigators' perspective. This tool includes 22 items: 4 items related to patients, 5 to centers, 5 to surgeons and 8 to the intervention. This tool aims at describing the items that should be considered before applying the results of surgical trials in clinical practice. Of course, the interpretation of the applicability of the research results by readers of this tool implies keeping in mind that the decision to implement or not the results into clinical practice depends highly on the level of evidence of the research.

Understanding the determinants of the applicability of results of an RCT requires clinical rather than statistical expertise [11]. Consequently, the investigators and other care providers who participated in the trial should be in a good position to adequately appraise and depict the applicability of the results of their own trial. The tool should be used similar to reporting guidelines such as the CONSORT checklist [13]. This usage supposes that investigators and authors of manuscripts change their behavior and agree to complete the tool. As for all reporting guidelines, this tool will need to be endorsed and implemented by journal editors.

This new graphical tool should have several advantages. First, it is the first tool allowing for an appraisal and depiction of the applicability of results of a surgical trial. Second, the appraisal results from consensus of a multidisciplinary team, which allows for taking into account different perspectives in the assessment of applicability. This process is particularly useful to take into account the important subjectivity of the assessment of applicability of a trial's results. Third, the applicability will be assessed by people participating in the trial and consequently having a clear understanding of how the trial was actually planned and conducted. Fourth, the assessment will not depend on the adequate reporting of all the data necessary to appraise the applicability. Fifth, the tool could also have a pedagogical impact in that it will force investigators to question the applicability of their results. Finally, this tool will overcome the issue of the poor quality of reporting in surgery articles [14], [15]: the investigator will be transparent and present the assessment of applicability using a specific figure. This presentation will allow reviewers and other experts to challenge authors about their assessment before or after publication.

This new tool also has some limitations. First, the search strategy to identify external validity items was not exhaustive; however, surgeons had the opportunity to add important items. Second, some items may be missing, such as the country of practice of investigator, which can be particularly important in some contexts. However, the investigator can add some new items in the tool according to their context. Third, this tool provides for an assessment and depiction of the applicability of surgical RCT results at the time of their publication and may need an updated assessment with time. In fact, the notion of applicability is a dynamic concept that changes with time. A novel technical procedure performed only in a super-specialized center will not have the same level of applicability 10 years later, when the practice of surgeons, their habits and the infrastructures of centers have adopted this new technique. Surgeons may have difficulty taking into account what they already know about the intervention when learning new information from a study report. For example, endoscopic surgery for cholecystectomy was originally a confidential technique performed by expert surgeons in specialized centers. Several years later, it became a widespread technique in most centers performing gastrointestinal surgery. Further investigators completing the tool may have some conflict of interest or particular hunches related to the applicability of their trial results. The use of this tool will force authors to clarify their position and to allow readers, reviewers and editors to challenge their appraisal. Further, the reporting of authors' appraisals does not imply that readers should follow the authors' recommendations. Fourth, the reproducibility of the tool is not optimal. Therefore, the assessment of applicability must result from a consensus of different investigators participating in the trial. Fifth, the quality of the depiction of the applicability of the trial will depend on the quality of the process used to appraise the applicability. Therefore, complete transparency in the process is essential, and the figure must clearly indicate whether it represents the assessment of one surgeon or several surgeons, physicians, anesthetists, for example. In fact, readers will probably have less confidence if the figure represents only one investigator's belief but more confidence if it represents the consensus of several investigators with varied expertise. If no consensus is obtained, this could be specified in the article. Sixth, the process to complete this tool could be considered time-consuming. However, the process requires only that each investigator complete the checklist, which takes less than 10 minutes, and to organize a 1-hour meeting to obtain consensus. To help investigators complete the tool, we will provide free access to a web-based program that will allow for automatic collection of investigators' assessments and automatic construction of the figures. Further, the effort required to use this tool should be weighed in terms of the importance of providing a clear assessment of the applicability of trial results. Finally, the time necessary to complete this process is minimal in light of the time and energy spent planning the trial; obtaining funding; recruiting, treating, and following patients; performing data entry, management, and analysis; and writing the manuscript.

Some other authors have highlighted the need to develop new tools to appraise the applicability of trial results. For example, Perera and colleagues have proposed graphical methods for depicting RCTs of complex interventions to clarify the basic structure of the experimental and control intervention [16]. Several authors have proposed a checklist to appraise the applicability of results in different contexts [12], [17]. However, to our knowledge none of these tools were an investigator-based assessment of the applicability of trial results. Finally, Thorpe and colleagues developed the pragmatic-explanatory continuum indicator summary (PRECIS) [18], [19]. This tool aims to help trialists appreciate the degree to which their trial is a pragmatic or an explanatory trial when planning their trial. Although the concepts of pragmatic/explanatory and applicability are very close, the purpose of our tool clearly differs.

Our study has several limitations. First, the representativeness of the RCTs evaluated could be questionable. We evaluated the applicability of only one-third of the surgical RCTs with published results and indexed in 2009. Second, we focused on only surgeons' evaluations. The appreciation of applicability by clinicians such as anesthetists could be different. Therefore, the consensus process should involve investigators with different expertise (surgeons, anesthetists). Third, the threshold to decide to include an item in the final checklist could seem arbitrary. However, developing a checklist with this method always involves use of an arbitrary threshold. We did not choose a more stringent threshold (more than 50%, or only items considered as essential) because we felt that we would exclude important items that might require attention in half of published or ongoing trials and would not be in the tool. To counterbalance this arbitrary choice, this tool should be tailored for each trial. For example, if an item considered important by investigators is not in the tool, the investigators should add it. Similarly, if an item in the tool is not relevant for the trial results being evaluated, the item could be deleted. Fourth, applicability is a complex and multidimensional concept. Its interpretation is difficult and could depend on the conclusions of the RCT. We included all reports of surgical RCTs in our survey without taking into consideration the trial results. An outcome not favoring the surgical procedure tested may have modified the interpretation of the applicability of results. Further, surgeons may undereestimate the importance of some items, particularly those related to patients and centers. In our survey, surgeons considered co-morbidities and co-medications of patients essential for only 13% of surgical RCTs. Finally, the impact of this tool will need to be validated in further studies.

In conclusion, the issue of the applicability of results is important to consider for surgical trials. We developed a tool assessing and depicting the applicability of results of a surgical RCT according to the investigators' perspective. This new tool could 1) help the reader judge what needs attention before applying the results of a surgical RCT to their own clinical practice [20], [21] and 2) help researchers, systematic reviewers, and investigators discuss and criticize the applicability of the RCT results.

## Supporting Information

Text S1 **The search strategy to identify the most relevant items.**
(DOCX)
