# Supplementary material for: ASSIST Applicability Scoring of Surgical trials. An Investigator-reported aSsessment Tool
Source: PLoS One. 2012 Aug 15;7(8):e42258. doi: 10.1371/journal.pone.0042258 (PMC3419723; doi:10.1371/journal.pone.0042258)
Supplement: Text S1 — The search strategy to identify the most relevant items. (DOCX) [file pone.0042258.s001.docx]

**Text S: The search strategy to identify the most relevant items**

**Medline via PubMed.**

MeSH terms and free text were used to identify studies, with no limit on time.

Search done the 02/2010

"external validity"[Text Word]

generalisability[Text Word] AND validity[Text Word] (19 duplicates)

(applicability[Text Word] ) AND validity[Text Word] (40 duplicates)

generalisability[All Fields] (122 duplicates)

(generalizability[Text Word]) AND validity[Text Word] (100 duplicates)

"external validity"[All Fields] AND ("surgery"[Subheading] OR "surgery"[All Fields] OR "surgical procedures, operative"[MeSH Terms] OR ("surgical"[All Fields] AND "procedures"[All Fields] AND "operative"[All Fields]) OR "operative surgical procedures"[All Fields] OR "surgery"[All Fields] OR "general surgery"[MeSH Terms] OR ("general"[All Fields] AND "surgery"[All Fields]) OR "general surgery"[All Fields]) ( 106 duplicates)

external[All Fields] AND validity[All Fields] AND ("surgery"[Subheading] OR "surgery"[All Fields] OR "surgical procedures, operative"[MeSH Terms] OR ("surgical"[All Fields] AND "procedures"[All Fields] AND "operative"[All Fields]) OR "operative surgical procedures"[All Fields] OR "surgery"[All Fields] OR "general surgery"[MeSH Terms] OR ("general"[All Fields] AND "surgery"[All Fields]) OR "general surgery"[All Fields]) ( 111 duplicates)

| Most Recent Queries | Result |
| --- | --- |
| Search generalisability | [484](http://www.ncbi.nlm.nih.gov.gate2.inist.fr/pubmed/?cmd=HistorySearch&querykey=5&) |
| Search generalisability[Text Word] AND validity[Text Word] | [112](http://www.ncbi.nlm.nih.gov.gate2.inist.fr/pubmed/?cmd=HistorySearch&querykey=4&) |
| Search (applicability[Text Word] ) AND validity[Text Word] | [1158](http://www.ncbi.nlm.nih.gov.gate2.inist.fr/pubmed/?cmd=HistorySearch&querykey=3&) |
| Search external validity | [3192](http://www.ncbi.nlm.nih.gov.gate2.inist.fr/pubmed/?cmd=HistorySearch&querykey=1&) |
| Search "external validity"[Text Word] | [1254](http://www.ncbi.nlm.nih.gov.gate2.inist.fr/pubmed/?cmd=HistorySearch&querykey=1&) |
|  |  |
| Search (generalizability[Text Word]) AND validity[Text Word] | [553](http://www.ncbi.nlm.nih.gov.gate2.inist.fr/pubmed/?cmd=HistorySearch&querykey=2&) |
| Search "external validity" surgery | [106](http://www.ncbi.nlm.nih.gov.gate2.inist.fr/pubmed/?cmd=HistorySearch&querykey=3&) |
| Search external validity surgery | [254](http://www.ncbi.nlm.nih.gov.gate2.inist.fr/pubmed/?cmd=HistorySearch&querykey=4&) |

**The Cochrane Methodology Register.**

MeSH terms and free text were used to identify studies, with no limit on time.

Search done the 02/2010

"external validity” search all text

Cochrane Methodology Register (Methods Studies)

There are **91** results out of **12778 records**

Inclusion : 0 articles
